# Stuttering Speech

**Published:** 1888-11

**Authors:** 


### STUTTERING SPEECH.

### Some Addenda by S. L.

When we ask, What is stammering and stuttering? Kussmaul, in his great work, *The Disturbances of Speech*, answers it thus: Stammering consists in the loss of the faculty to pronounce certain letters correctly, while stuttering is a spasmodic loss of power, coming off and on, to vocalize sounds, especially the explosive consonants. Though the stutterer utters correctly every single letter, he cannot connect them to syllables on account of the difficulty to vocalize them. We may say, therefore, that the *co-ordination of the muscular motion for letters is another function, and arises from different central apparatus than that for syllables and words.* Stuttering is a spastic neurosis of co-ordination; it is a dysarthria syllabasis, appearing at certain times and under certain circumstances, while in aphthongia spasms appear in the hypoglossus at every attempt to speak, and thus speaking becomes an impossibility. Stammering, dysarthria literalis balbuties, may be congenital or acquired, functional from wrong education and deficient exercise, or from organic causes in central parts of the nervous system, or localized in the motory nerves of speech, especially in the hypoglossus. Thus Kussmaul gives us two valuable hints. We have to look at the remedies for inco-ordination, a kind of localized chorea of speech, if I may say so. We deal here with a neurosis plain and simple, and it is a well-known fact that the longer this neurosis lasts, the more it becomes a habit, the more obstinate will it be to eradicate the vice. We have, therefore, a wider scope when we look for disturbances of speech than only for stammering and stuttering, and with due respect to our good friend, the editor, we would wish to lead his attention to some other drugs which also show disturbances of speech, as:

*Anacardium.*—When speaking he finds it difficult to utter certain words, as if his tongue were too heavy; great mental weakness; he fails to know what and how to say it; heaviness of tongue, and sensation as if swollen; impeded speech,

*Artemisia vulg.*—Speech unintelligible; can utter but single words, and these only with great exertion; froth at the mouth.

*Argentum nitr.*—Speech stammering, cannot talk from spasms of the muscles of tongue and throat; ptyalism; limbs, especially his knees, start up at night, awaking him; hands tremble; foul breath.

*Asafœtida.*—Neuroses of hysterical and scrofulous people; speech unintelligible; tongue white, swollen; frequent muscular jactitations in arms and legs; constantly chewing and working frothy slime out of his mouth.

*Calcarea ostr.*—Speech is difficult and clumsy; tongue pushed upward and to the left; copious flow of viscid saliva; ravenous hunger, with weak stomach; bulimy in the morning; trembling motion of upper and lower limbs in spells.

*Causticum.*—Stuttering, difficult, indistinct speech; muscles of tongue affected so that speech is thick and words are jerked out; salivation; ravenous hunger, takes food in a hurried manner; twisting and jerking of limbs.

*Cicuta.*—Speech difficult from having no control over movements of mouth and tongue; great hunger shortly after a meal; irresistible desire to eat coal; jerking of limbs.

*Crocus sat.*—Absence of mind and forgetfulness, makes constant mistakes in words; music is the only thing which clings to his mind.

*Kali brom.*—Disturbance of speech, emanating from brain, medulla, or spinal cord; action of tongue disordered; stammering; slow and difficult after waking; profuse saliva, with fœtid breath; fitfulness of motion, must be on the move.

*Lac caninum.*—Difficulty in articulating, owing to a paretic state of tongue, causing stuttering if she talks fast, has to speak very slowly; mouth full of frothy saliva.

*Lachesis.*—Stammering, letters s, b, t, w; stammering comes with second or third word or not in a whole period; saliva abundant and tenacious; hunger, cannot wait for food; jerking of extremities with restlessness.

*Laurocerasus.*—Indistinct speech and gets angry when not understood; unusual appetite.

*Lycopodium.*—Cannot read, because he mistakes letters; he can write correctly, but cannot read what he wrote, leaves out syllables and cannot find the right word for common things; tongue stiff; stuttering without any appreciable cause.

*Platina.*—Stuttering, her voice sounds as if she had something in her mouth, as if the posterior organs of speech were covered and clumsy; hysterical disturbances of speech.

*Spigelia.*—Repeats the first syllable of the first word several times, after that speaks plainly; helminthiasis.

*Zincum.*—Echo speech; patients repeat in a monotonous, singing way the words and the sentences of their neighbor without being conscious of it; weakness of the organs of speech when reading. (Tabacum.)

With the remedies mentioned by Dr. Lee, page 545 of this journal, we are certainly not without means to battle successfully with these disturbances of speech. But let us not be absorbed entirely by looking up the simillimum; there are other and equally important aids at our command, for Kussmaul insists on careful nutrition, hydropathy, gymnastics of the lungs, and careful watchfulness, so that the patients may pronounce every syllable clearly, slowly, and distinctly, when we want to cure successfully their ailments. The patient must learn to use his will-power, and silence in company, especially during exciting conversations, cannot be too highly recommended.

